# Origins, Phytochemistry, Pharmacology, Analytical Methods and Safety of Cortex Moutan (*Paeonia suffruticosa* Andrew): A Systematic Review

**DOI:** 10.3390/molecules22060946

**Published:** 2017-06-07

**Authors:** Zhiqiang Wang, Chunnian He, Yong Peng, Feihu Chen, Peigen Xiao

**Affiliations:** 1 School of Pharmacy, Anhui Medical University, Hefei 230032, China; wzqwillis1016@gmail.com; 2 Institute of Medicinal Plant Development, Chinese Academy of Medical Science, Peking Union Medical College, Beijing 100193, China; ypeng@implad.ac.cn (Y.P.); xiaopg@public.bta.net.cn (P.X.); 3 Key Laboratory of Bioactive Substances and Resources Utilization of Chinese Herbal Medicine, Ministry of Education, Beijing 100193, China

**Keywords:** Cortex Moutan, origins, phytochemistry, pharmacology, safety, Traditional Chinese Medicine

## Abstract

Cortex Moutan (CM), a well-known traditional Chinese medicine, is commonly used for treating various diseases in China and other eastern Asian countries. Recorded in Pharmacopeias of several countries, CM is now drawing increasing attention and under extensive studies in various fields. Phytochemical studies indicate that CM contains many valuable secondary metabolites, such as monoterpene glycosides and phenols. Ample evidence from pharmacological researches suggest that CM has a wide spectrum of activities, such as anti-inflammatory, anti-oxidant, anti-tumor, anti-diabetic, cardiovascular protective, neuroprotective, hepatoprotective effects. Moreover, various analytical methods were established for the quality evaluation and safety control of CM. This review synopsizes updated information concerning the origins, phytochemistry, pharmacology, analytical method and safety of CM, aiming to provide favorable references for modern CM research and application. In conclusion, continuing pharmacological investigations concerning CM should be conducted to unravel its pharmacological mechanisms. Further researches are necessary to obtain comprehensive and applicable analytical approach for quality evaluation and establish harmonized criteria of CM.

## 1. Introduction

Traditional Chinese Medicine (TCM) plays an indispensable role in the healthcare system of Chinese people due to its efficiency for various diseases, and still contributes to satisfy the modern medical demand owing to its large-scale compounds reservoir for new drug discovery. Cortex Moutan (CM), dried root bark of *Paeonia suffruticosa* Andrew (Fam. Ranunculaceae/Paeoniaceae), is an important crude drug traditionally used in China to treat diverse diseases for thousands of years [1]. *P. Suffruticosa*, called “Mudan” in Chinese vernacular names, is equally famous for its ornamental and medicinal uses: its flower is a symbol of elegance and prosperity, while its root bark, namely “Mudanpi” in Chinese, is broadly used in TCM as remedies for cardiovascular, extravasated blood, stagnated blood, and female genital diseases [2]. CM was recorded in several famous Chinese medical books, like *Compendium of Materia Medica* (*Ben Cao Gang Mu*), *Shen Nong's Herbal Classic* (*Shen Nong Ben Cao Jing*) and *Chinese Materia Medica* (*Zhong Hua Ben Cao*), etc. In addition, it is widely used in eastern Asian countries and recorded in Pharmacopeias of several countries, such as China, Japan, Korea and Vietnam. In the theory of TCM, it is believed that CM alleviates sickness in humans by clearing excessive heat, cooling the blood, promoting blood circulation, and removing blood stasis without inducing bleeding. CM was applied in a great number of prescriptions, exemplified as “Liuwei Dihuang wan” for yin deficiency; “Guizhi Fuling wan” and “Wen Jin tang” for chronic female diseases; “Ba Wei tang” for disease of the aged, like diabetes and arteriosclerosis; and “Dahuang Mudan tang” for appendicitis and carbuncles [3]. There are 79 CM containing preparations in Chinese Pharmacopoeia (2015 edition). Moreover, CM was found in 13 formulations recorded in Taiwan Herbal Pharmacopoeia (Second edition, Chinese version) and 8 commonly used kampo medicines [4].

Given its wide application, CM is now being extensively studied in phytochemistry, pharmacology and chemical analysis. Last several decades have saw plethora of papers reporting isolation and identification of many components in CM extracts, notablely paeonol, paeoniflorin, paeonoside, apiopaeonoside, oxypaeoniflorin, galloylpaeoniflorin, galloyloxypaeoniflorin, mudanpioside A, B, C, D, E, H, suffruticoside A, B, C, D, E, benzoyloxypaeoniflorin, benzoylpaeoniflorin and gallic acid, etc. [5,6]. Meanwhile, it is reported that CM extracts have a wide spectrum of pharmacological activities, including anti-inflammatory [7,8], anti-allergic [9,10], and anti-oxidative effects [11,12]. For the chemical analysis of CM, many qualitative and quantitative methods, such as high performance liquid chromatography (HPLC), gas chromatography (GC), capillary electrophoresis (CE), and liquid chromatography tandem mass spectrometry (LC-MS), are established for the comprehensive quality evaluation of CM. HPLC methods are often established for multi-components assaying simultaneously, and LC-MS methods greatly support unambiguous identification and high sensitive quantifications of compounds at trace concentrations.

Nevertheless, as herbal medicine with complicated compounds, quality evaluation and quality control of CM remain challenging for modern researchers and TCM practitioners. In China, CM was produced from different origins with different processing methods. Therefore, discrepancies in chemical composition of different CM may exist. Moreover, in the general market, CM is only graded by various aspects of their physical appearances, such as root length and diameter. Besides, confusion remains about the pharmacological mechanism of CM, as therapeutic effects of TCMs often come as a result of synergistic effects of multi-compounds. However, quality criteria for CM in Chinese Pharmacopeia and other East Country Pharmacopoeias rely on only a single or a few constituents as chemical makers, such as paeonol and paeoniflorin. In fact, only detecting paeonol and paeoniflorin is at best partial and inadequate to reflect the holistic quality of CM.

This review intent to compile various researches and critically summarize the issues related to origins, phytochemistry, pharmacology, analytical methods and safety about CM. The isolated bioactive constituents and reported biological activities of CM over the past few decades are synopsized. Analytical methods concerning CM in recent years are also outlined. Overall, the aim of this review is to provide favorable references for the modern application and research of CM, such as quality control, quality evaluation and standard improvement during production and processing, etc.

## 2. Origins of CM

**Figure 1:**
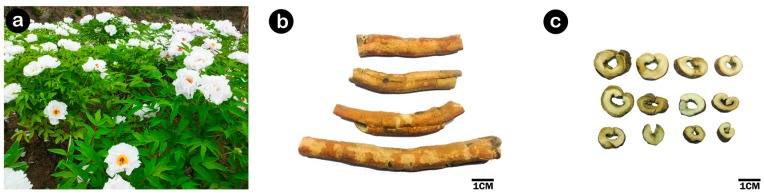
(**a**) Plants of *P. suffruticosa*; (**b**) Crude drug of *Guadanpi*; (**c**) Decoction pieces of Cortex Moutan (CM).

According to Chinese Pharmacopoeia, the original plant of CM is always considered to be *P. suffruticosa* Andrews, which is the collective name of cultivated tree peonies [13]. Recently, as the botanists further refine the taxonomy, section *Moutan* DC of the genus *Paeonia* L. in the family Paeoniaceae were generally subdivided into nine wild shrubby species: *P. cathayana*, *P. decomposita*, *P. jishanensis*, *P. ostii*, *P. qiui*, *P. rockii*, *P. rotundiloba*, *P. delavayi* and *P. ludlowii* [14]. Based on the botanist’s view, cultivated tree peonies, originated from the hybridization of multiple species of wild tree peonies, belong to *P. suffruticosa* complex. Besides, the cultivated *P. ostii* is also widely grown and considered major source of CM. Therefore, successive version of Chinese Pharmacopoeia regulate that the original plant for CM is *P. suffruticosa*(*s.l.*), which includes *P. ostii* and *P. suffruticosa* [15]. *P. suffruticosa*(*s.l.*) is mainly cultivated in different areas with different vernacular names: CM in Tongling (Anhui province) is called “Feng Danpi”, CM in Dianjiang (Sichuan province) is called “Chuan Danpi” and CM in Heze (Shandong province) is called “Cao Danpi”. In addition, root cortex of several wild tree peonies, such as *P. decomposita*, *P. delavayi*, *P. rockii*, *P. jishanensis*, are used as substitute of CM due to morphological similarity in different regions of China. For example, root cortex of *P. delavayi* Franch, called “Diandanpi”, is often used in Yunnan province as a folk medicines substituting CM. In general, the root of *P. suffruticosa*(*s.l.*) is often collected in autumn, removed from rootlets and soil, and then manufactured into two forms of official drug of CM, *Liandanpi* and *Guadanpi*, with different process method respectively. In the former method the root bark is stripped off, and dried in the sun, while in the latter method scrape off tertia, removed from duramen then dehydrated in the sun [13] (Figure 1).

## 3. Chemistry of CM

**Figure 2:**
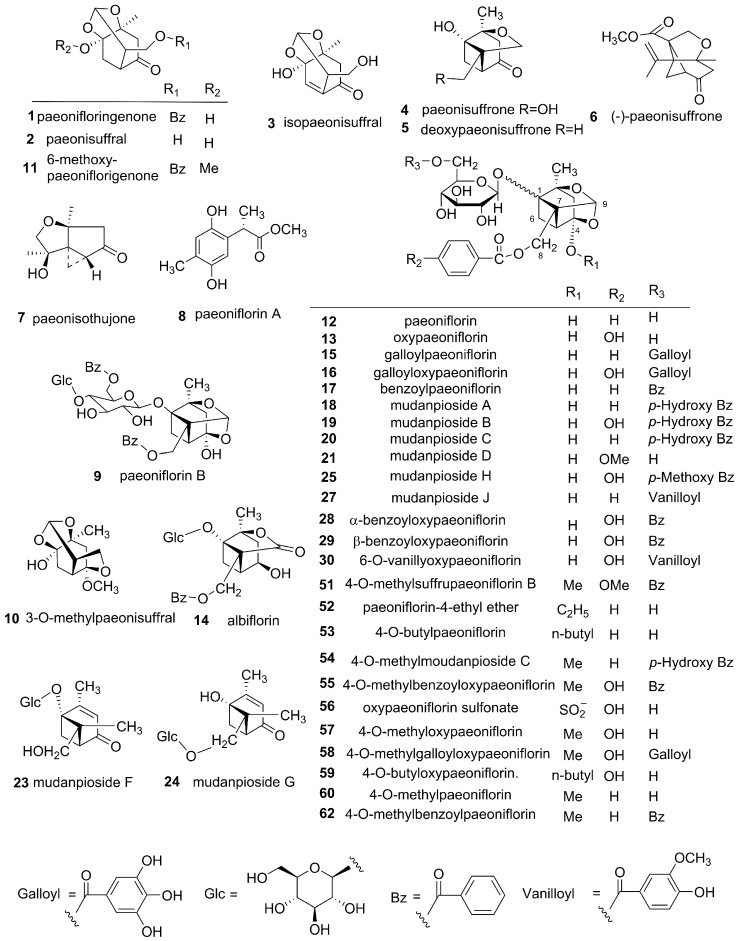

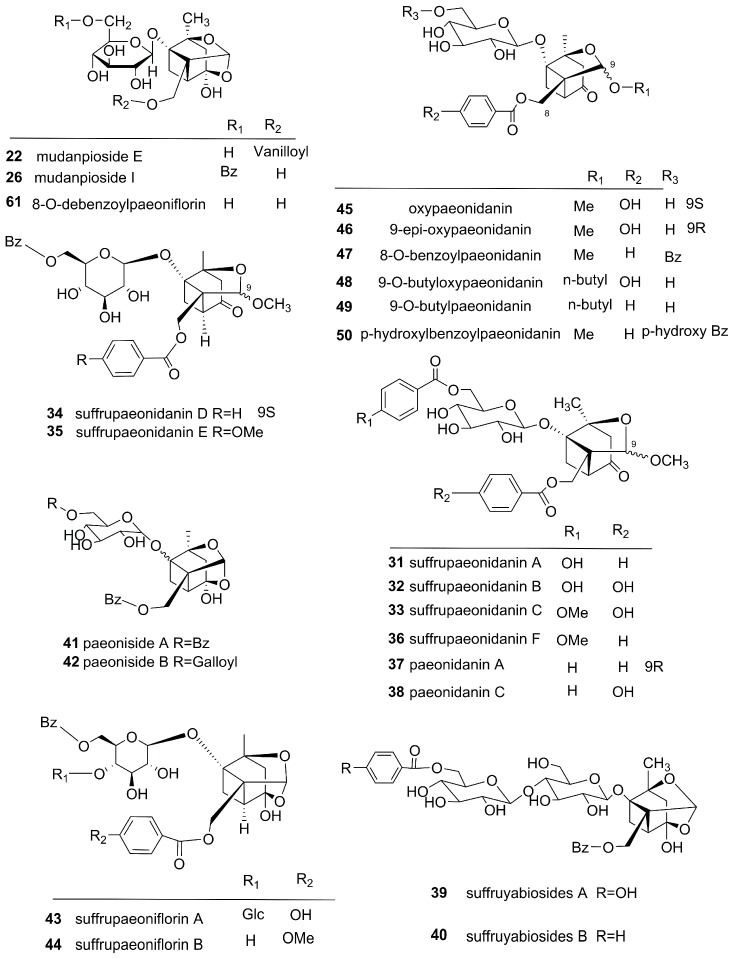

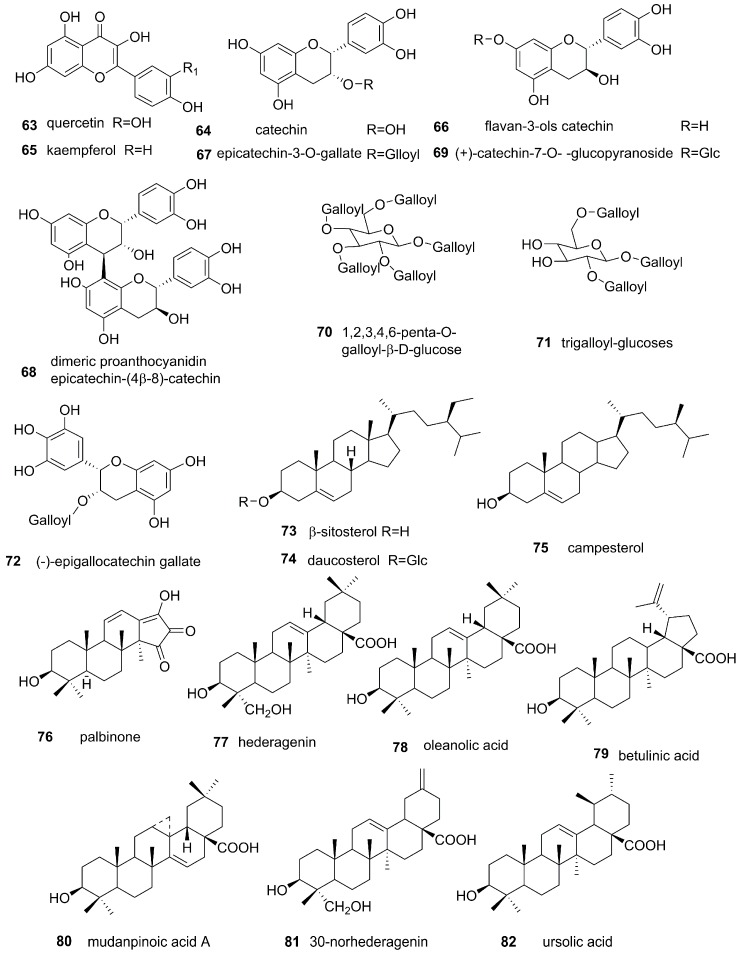

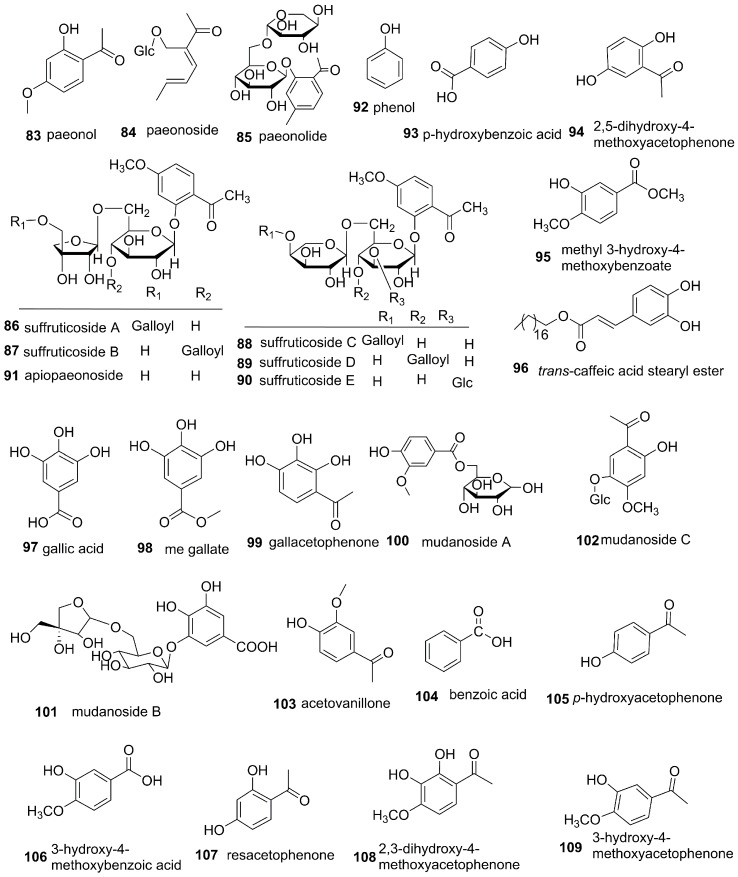

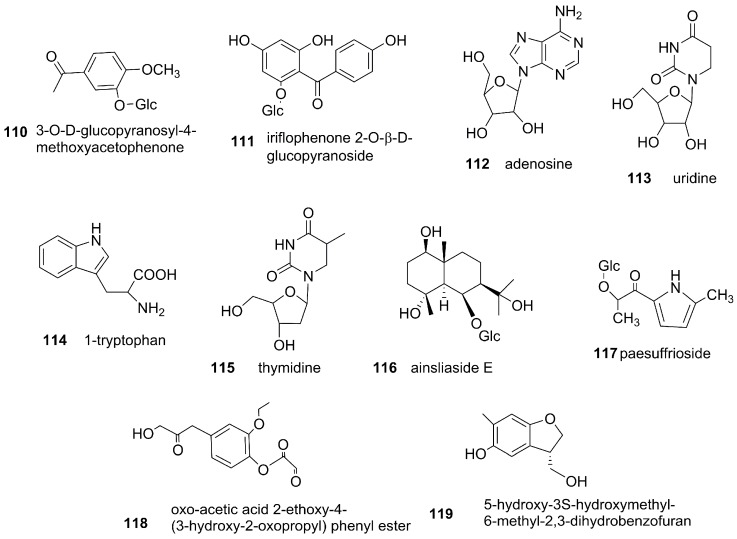
Chemical structures of 119 compounds isolated from CM.

To our current best knowledge, 119 compounds have been isolated and structurally identified from CM, which can be assigned to seven classes: monoterpenes, monoterpene glycosides, flavonoids, tannins, triterpenoids, phenols and others [16,17,18,19,20,21,22,23,24,25,26,27,28]. Monoterpene glycosides and phenols are predominant constituents in CM (Figure 2 and Table S1).

### 3.1. Monoterpenes and Monoterpene Glycosides

A total of 10 monoterpenes, **1**–**8**, **10**, **11**, and 52 monoterpene glycosides, **9**, **12**–**62**, were reported from CM. The absolute stereostructures of paeonisuffrone (**4**), paeonisuffral (**3**), paeonisothujone (**7**), deoxypaeonisuffrone (**5**) and isopaeonisuffral (**3**) have hitherto been reported [29,30]. Paeonisothujone (**7**) is the first natural example of ortho-menthane-type monoterpene having a cyclopropane ring and paeonisuffrone (**4**) is a tricyclic compound [31].

Monoterpene glycosides, such as paeoniflorin (**12**) and its analogues **13**–**62**, were ubiquitous chemical components across all species of the genus *Paeonia* which possesses a “cage-like” pinnae skeleton. Compounds **13**–**62** are pinnae type derivatives resembled closely to each other, the common pattern is a pinnae skeleton with a aglycone and one or two different moieties with a variety of substituent groups, like benzoyl, galloyl, *p*-hydroxybenzoyl, vanilloyl, etc. For example, mudanpioside A–E (**18**–**22**) were mono- or di-benzoates of monoterpene glycosides. The difference between them lies in the substitution pattern of the aromatic rings [32]. Recently, new monoterpene glycosides are continuingly reported, such as paeoniside A (**41**) and paeoniside B (**42**). Paeoniside A (**41**) has a monoterpene system and same aglycone of paeoniflorin (**12**) togother with two benzoyl moieties, while the structure of paeoniside B (**42**) was very similar to paeoniside A (**41**), except for the absence of a benzoyl moiety and the appearance of galloyl moiety [33]. It was reported in 2012 that suffruyabiosides A and B (**39**,**40**) were rare two new monoterpene diglycosides with a cellobiose in the molecules [34]. Among all the identified monoterpene glycosides, several pairs of isomers were found. For instance, α-benzoyloxypaeoniflorin (**28**) and β-benzoyloxypaeoniflorin (**29**), benzoylpaeoniflorin (**17**) and paeoniside A (**41**) are α- and β-anomers, respectively. Suffrupaeonidanin D (**34**) and paeonidanin A (**37**), oxypaeonidanin (**45**) and 9-epi-oxypaeonidanin (**46**) are unambiguously confirmed chiral isomers, respectively.

### 3.2. Flavonoids and Tannins

To date, seven flavonoids **63**–**69** and three tannins **70**–**72** were obtained from CM. Flavonoids reported from CM are quercetin (**63**), kaempferol (**65**), catechin (**64**) and catechin derivatives (**66**–**69**). Tannins found in CM are galloyl glucoses, they are 1,2,3,4,6-Penta-*O*-galloyl-β-d-glucose (PGG, **70**), trigalloyl-glucoses (**71**) and (−)-Epigallocatechin gallate (**72**) [35,36,37].

### 3.3. Phenols

A total of 29 phenols were isolated from CM. Phenols **83**–**111**, especially acetophenones (**99**, **105**, **107**–**110**), are the characteristic metabolites mainly reported from *P. suffruticosa* and present in *P. albiflora* and *P. lactiflora* in scarcely low levels. Paeonol (**83**) and paeonol glycosides, like paeonoside (**84**), paeonolide (**85**), apiopaeonoside (**91**) and suffruticoside A–E (**86**–**90**), are characteristic and major components in CM. Some of the phenols, such as gallic acid (**97**), benzoic acid (**104**) are distributed widely in *Paeonia*. Recently, several new phenols were reported from *P. suffruticosa*, they are mudanoside C (**102**), iriflophenone 2-*O*-β-d-glucopyranoside (**111**) [38].

### 3.4. Triterpenoids and Others

So far, 10 triterpenoids, **73**–**82**, and other compounds, **112**–**119** have been reported from CM. **73**–**76** are tetracyclic triterpenoids whereas **77**–**82** are pentacyclic triterpenoids. Other compounds, like adenosine (**112**), uridine (**113**), 1-tryptophan (**114**), thymidine (**115**), ainsliaside E (**116**), and paesuffrioside (**117**) were reported to be present in CM water-soluble constituents [39].

## 4. Pharmacological Activities of CM

### 4.1. Anti-Oxidative Effects

Reactive oxygen species (ROS) plays a central role in causing various types of diseases. The mechanism of CM to inhibit ROS production was studied intensively. The EtOH extract of CM inhibited the production of ROS on oxidative-stressed PC12 cells [11]. Besides, it was reported that total phenolic contents in methanol extracts of CM possessed significant antioxidant capacities and thus could be potential rich sources of natural antioxidants [40]. A significant relationship between antioxidant capacities and total phenolic contents were found, indicating that phenolic components are major contributor of antioxidant activities in CM. Paeonol (**83**), the predominant phenolic compound in CM, was reported to possess a variety of therapeutic properties by virtue of its free radical scavenging properties, for instance, paeonol (**83**) improved antioxidant defense system through the activation of Nrf2 related pathway in isoproterenol-induced myocardial infarction model [41] and attenuated cigarette smoke-induced lung inflammation via its antioxidant function and an inhibition of the MAPKs/NF-κB signaling [42].

Furthermore, paeoniflorin (**12**), a well-known extracellular ROS scavenger, exerts cytoprotective effects against ^6^^0^Co-ray-induced oxidative damage in thymocytes [43] and protects EA.hy926 cells against radiation-induced injury through the Nrf2/HO-1 pathway [44], indicating that paeoniflorin (**12**) offers a potential application in treating radiation-induced injury. Literatures also suggest that paeoniflorin (**12**) protects retinal pigment epithelium cells from oxidative stress [45]. Moreover, Galloylpaeoniflorin (**15**), galloylated derivate of paeoniflorin, showed cytoprotective effects against hydrogen peroxide (H_2_O_2_)-induced cell injury and death in human HaCaT keratinocytes [46]. Above all, ingredients of CM can significantly alleviate oxidative stresses and decrease ROS production. However, the above studies are largely carried on different cell lines, animal model or clinical tests are required in future investigations.

### 4.2. Anti-Inflammatory Effects

Published reports consistently demonstrate that CM possesses anti-inflammatory effects. Several in vivo and in vitro model stimulated by lipopolysaccharides (LPS) have been developed to study the anti-inflammation effect of CM and its principal components. For example, administration of CM prior to LPS challenge improves acute lung injury mediating through anti-inflammation in rat models [2]. And CM has anti-inflamamatory effects through the inhibition of iNOS and COX-2 expression by suppressing the phosphorylation of I-κBα and the activation of NF-κB in LPS-Activated macrophage cells [47]. The expression levels of LPS-induced genes in macrophages were altered, to different extents, by treatment with paeonol (**83**), paeoniflorin (**12**), and albiflorin (**14**) [48]. Moreover, inflammatory changes of gene expression in LPS-stimulated gingival fibroblasts was studied using a genome-wide expression GeneChip, results suggest CM inhibits the induction of inflammation by comprehensively inhibiting a wide variety of activations of inflammation-related genes, which may be due to paeonol (**83**) and paeonoflorin (**12**) [8]. Besides, paeoniflorin (**12**) was proven to exert anti-inflammatory effect in animal models of collagen-induced arthritis, ischemia/reperfusion-induced cerebral injury, LPS-induced acute lung injury and liver inflammatory reactions. For example, paeoniflorin (**12**) inhibits LPS-induced inflammation in human umbilical vein endothelial cells concomitantly with decreased expression of the enhanced high mobility group box-1 (HMGB1), downregulated mRNA and protein expression of RAGE, TLR-2 and TLR-4, and decreased NF-κB activity [49].

CM can significantly inhibit the secretion of inflammatory chemokines in several cell lines and a rat model. Methanolic extract of CM, specifically PGG (**70**), markedly suppressed secretions of IL-8 and macrophage chemo-attractant protein-1 in human monocytic U937 cells stimulated with phorbol myristate acetate [50]. Paeoniflorin (**12**), paeonol (**83**), and PGG (**70**) exhibited dose-dependent inhibition of TNF-α synthesis and IL-6 production in synoviocytes treated with pro-inflammatory mediator [7]. Besides, it was reported that paeonol (**83**) suppressed LPS-induced inflammatory cytokines in macrophage cells and protected mice from lethal endotoxin shock. In vitro study suggests paeonol (**83**) down regulated the production of TNF-α, IL-1β, IL-6, and IL-10 via inactivation of I-κBα, ERK1/2, JNK, and p38 MAPK. Moreover, paeonol (**83**) significantly regulates pro- and anti-inflammatory cytokines in mouse model of LPS-induced endotoxemia [51]. However, bioavailability of these identified compounds are not good enough; it can be anticipated that new synthetic agents derived from active compounds in CM can have good bioavailability.

### 4.3. Anti-Tummor Effects

Various researches have been conducted on anti-tumor effects of CM in recent years. Antiproliferative effects of CM on human cancer cell lines encompasses several common malignancies, such as breast ductal carcinoma, colon cancer, hepatocellular carcinoma, gastric cancer, and esophageal cancer [52,53]. CM extract blocks the binding of vascular endothelial growth factor (VEGF), an important angiogenic molecule, to VEGF receptor and reduce VEGF-induced endothelial cell proliferation [54]. As we know, angiogenesis plays a critical role in tumor growth and metastasis processes. It is suggested that CM may be used as a candidate for developing anti-angiogenic agent. Researches also revealed that CM exhibited high selectivity in inhibiting the growth of bladder cancer cells and reduced the expression of angiogenesis-stimulating factors, including VEGF [55].

Recently, several compounds involved in anti-tumor effects of CM were investigated. Paeonol (**83**) was reported to suppress chondrosarcoma metastasis [56] and melanoma metastasis [57]. Several studies indicate that paeonol (**83**) induces tumor cell apoptosis in HepG2 cells [58], mice bearing EMT6 breast carcinoma [59] and a HepA-hepatoma bearing mouse model [60]. Moreover, paeonol (**83**) reverses paclitaxel resistance in human breast cancer cells by regulating the expression of transgelin 2 [61] and exerts an anticancer effect on human colorectal cancer cells through inhibition of PGE2 synthesis and COX-2 expression [62]. Paeoniflorin (**12**) inhibits proliferation and invasion of breast cancer cells [63] and macrophage-mediated lung cancer metastasis [64]. In addition, paeoniflorin (**12**) inhibits proliferation and induces apoptosis of human glioma cells via microRNA-16 upregulation and matrix metalloproteinase-9 downregulation [65]. PGG (**70**) exhibits in vitro anti-proliferative effect on human hepatocellular carcinoma cell line, SK-HEP-1 cells [66]. In conclusion, paeonol (**83**), paeoniflorin (**12**) and PGG (**70**) cause no significant cytotoxic effects to normal cell lines, these compounds can be vital sources of adjuvant agent or complementary medicine during systemic chemotherapy in treating cancers.

### 4.4. Cardiovascular System Protective Effects

CM has been frequently used as an important ingredient in traditional prescriptions to relieve cardiovascular diseases, like Shuangdan granule, which has been authorized by SFDA of China to treat acute heart ischemia [67]. In TCM, CM has been commonly used to promote blood circulation and alleviate blood stasis. Nowadays, there is a growing awareness of the therapeutic potential of CM in cardiovascular system, and cardio-protective effects of CM are under extensive investigations. In a recent study, CM has been shown to protect the myocardium from ischemia/reperfusion injury by restoring the anti-oxidative defense system and increasing the expression of anti-apoptotic gene Bcl-2 [68]. Besides, paeonol (**83**) protects rat heart by improving regional blood perfusion during no-reflow [69].

The mechanism underlying the vasodilatatory effects of paeonol was investigated, an intracellular Ca^2+^ regulatory mechanism may be responsible for potent vasodilatory effect of paeonol [70]. Both paeonol (**83**) and paeoniflorin (**12**) have the potential to improve prethrombotic state and recanalize thrombi [71,72]. Furthermore, paeonol (**83**) has potential protective effects on the development of atherosclerosis through inhibiting oxidized low density lipoprotein-induced monocyte adhesion to vascular endothelial cell by inhibiting the mitogen activated protein kinase pathway [73]. Paeoniflorin (**12**) ameliorates acute myocardial infarction of rats by inhibiting inflammation and inducible nitric oxide synthase signaling pathways [74] and suppresses vascular damage and the expression of E-selectin and ICAM-1 in a mouse model of cutaneous Arthus reaction [75]. In summary, paeonol (**83**), paeoniflorin (**12**), benzoylpaeoniflorin (**17**), and α-benzoyloxypaeoniflorin (**28**) were found to be the major common active constituents and they would collectively contribute to improving blood circulation through their inhibitory effects on both platelet aggregation and blood coagulation. In addition, me gallate (**98**), catechin (**64**), paeoniflorigenone (**1**), galloylpaeoniflorin (**15**), and daucosterol (**74**) might also play a role in cardiovascular protective effects of CM [76]. More comprehensive animal and clinical studies should be conducted for the purpose of elucidating the therapeutic mechanism of CM on cardiovascular diseases.

### 4.5. Anti-Diabetic Activity

CM is a well-known herb found in anti-diabetic traditional medicine formulae, such as Liuwei Dihuang pills (LDP) [77]. Recently, scientific investigations about the extract of CM and its component are accumulating to explore its possible anti-diabetic mechanisms. Extraction of CM ameliorates the oxidative stress and inflammation in AGEs-induced mesangial cell dysfunction and streptozotocin (STZ)-induced diabetic nephropathy rats (DN) [12,78]. It is also reported that CM and its active component, especially paeonol (**83**), showed significant in vitro anti-diabetic effects by inhibiting glucose uptake of intestinal brush border membrane vesicles and enhancing glucose uptake into Hs68 and 3T3-L1 cells [79]. Furthermore, studies suggests paeonol (**83**) could improve the pathological damage of diabetic encephalopathy (DE) in STZ-induced diabetic rats through AGEs/RAGE/NF-κB pathway [80]. Paeoniflorin (**12**) has an anti-inflammatory effect in diabetic kidneys and prevents the development of nephropathy [81]. In addition, palbinone (**76**) and triterpenoids (**73**, **74**, **78**, **79**, **81**, **82**) remarkably stimulated glucose uptake and glycogen synthesis via AMPK pathway in a dose-depended manner. These compounds may have considerable potential for relieving the metabolic abnormalities associated with diabetic diseases [82]. In a word, most, if not all the active components of CM responsible for the hypoglycemic effect have been investigated and reported. CM can markedly improve glucose metabolism [83], attenuate diabetic syndromes like DE, DN and diabetic cataract [84].

### 4.6. Neuroprotective Activity

CM is now drawing increasing attention because of its neuroprotective activity. Many pharmacological investigations of CM have been addressed to elucidate the neuroprotective effects and underlying mechanisms of CM. According to previous studies, CM exhibits effectiveness in alleviating neuropathic pain [85] and neurodegenerative diseases, such as Parkinson disease [86]. Among compounds reported in CM, paeonol (**83**) and paeoniflorin (**12**) are well-known agents that have shown neuro-associated activities. Paeonol protected neurons from oxygen-glucose deprivation-induced injuries [87] and neurotoxicity caused by H_2_O_2_ treatment [88]. Moreover, another study implied that inhibition of NF-κB translocation to the nucleus and suppression of the mitogen activated protein kinase activities were involved in the anti-neuroinflammatory effects of paeonol (**83**) [89]. Paeonol (**83**) inhibits inflammatory and oxidative mediators in microglial cell through activation of AMPKα and GSK3α/β signaling pathway [90]. Also, paeonol (**83**) significantly improved cognitive deficit and neuropathologic lesion induced by D-gal injection in mice [91]. Following 6-hydroxydopamine toxicity in neuronal cells, paeonol (**83**) increases cell viability by inhibiting ROS production and increasing superoxide dismutase activity and Bcl-2 expression [92]. Treatment with paeonol (**83**) can protect against many of the alterations, including morphological, biochemical and behavioral changes, resulting from administration of Aβ_1__–42_ in a rat model of Alzheimer’s disease [93].

Recent investigations have demonstrated that paeoniflorin (**12**) administration can attenuate ischemia-induced cerebral injuries in rodent models [94] and alleviate glutamate or LPS-induced neuronal lesions [95]. Following glutamate, MPTP, and 6-hydroxydopamine toxicities, paeoniflorin (**12**) attenuates dopaminergic neuronal damage and behavioral impairments via the regulation of Bcl-2 family proteins and the inhibition of neuro-inflammation, in vitro and in vivo [96,97,98]. Moreover, paeoniflorin (**12**) protects neuronal cells from neurotoxins via an autophagic pathway and results in the degradation of α-synuclein [99]. Paeoniflorin (**12**) also inhibits 6-hydroxydopamine-induced apoptosis in PC12 cells via suppressing ROS-mediated PKCδ/NF-κB pathway [100]. Neuroprotective effects of paeoniflorin (**12**), but not the isomer albiflorin (**14**), are associated with the suppression of intracellular calcium and calcium/calmodulin protein kinase II in PC12 cells [101]. Besides, PGG (**70**) have strong inhibitory effects on formation of Aβ fibrils in vitro and in vivo [102] and protects rat neuronal cells (Neuro 2A) from hydrogen peroxide-mediated cell death via the induction of heme oxygenase-1 [103]. The current findings suggest that CM may be useful as alternative therapy to prevent and treat dopaminergic neuron dysfunctions [86].

### 4.7. Hepatoprotective Activity

Accumulating evidence indicates that CM has hepatoprotective activities. Pre-exposure of CM may attenuate acetaminophen-induced cytotoxicity through alleviation of GSH depletion, cytochrome P4502E1 activity, and hepatic DNA damage in vivo [104]. Paeonol (**83**) alleviates epirubicin-induced hepatotoxicity in 4T1-tumor bearing mice by inhibiting the PI3K/Akt/NF-κB pathway [105] and ameliorates alcoholic steatohepatitis in mice [106]. Pretreatment of paeoniflorin (**12**) protects mice against concanavalin A-induced hepatitis via inhibition of several inflammatory mediators and downregulation of the NF-κB pathways [107]. Besides, paeoniflorin (**12**) alleviates liver fibrosis by inhibiting HIF-1α through mTOR-dependent pathway [108]. Above all, CM is traditionally used as dietary supplement or TCM to treat hepatitis, and the above investigations may provide scientific explanations for the traditional application.

### 4.8. Others

Increasing studies suggest that CM possesses a broad range of other biological activities like anti-bacterial [109,110], anti-allergic [9,111], immunomodulatory [112], anti-fungal [113] and alleviating colitis [114]. Moreover, Paeoniflorin (**12**) promotes non-rapid eye movement sleep via adenosine [115].

Several studies were carried out to screen bioactive compounds in CM. In vitro experiment verified that paeoniflroin (**12**), PGG (**70**), and paeonol (**83**) reduced the activity of nicotinamide-adenine dinucleotide phosphate oxidase (NADPH) activity and decreased the level of ROS [116]. Similarly, in order to analyze the bioactive compounds in CM on treating nephropathy, mouse renal mesangial cells were cultured and used to bind and separate components in CM extraction. One compound which could interact with mesangial cells was found and identified as paeonol (**83**) [117]. In summary, CM has exhibited various pharmacological benefits; it can be a promising alternative or adjuvant therapy for various diseases.

## 5. Analytical Methods for Quality Evaluation of CM

### 5.1. Quality Criteria of CM in Different Countries

There are slight differences in the nomenclature and some aspects of the use of CM in different Pharmacopoeias, such as Chinese Pharmacopoeia, Japanese Pharmacopoeia, Korean Pharmacopoeia, Vietnamese Pharmacopoeia, Hong Kong Chinese Materia Medica Standards and Taiwan Herbal Pharmacopoeia [118]. Descriptions of CM, like length, diameter and thickness, vary from each other too. However, the testing methods and specification values for CM vary significantly in different pharmacopoeias. For instance, criteria for CM in Chinese Pharmacopoeia stipulate that the content of paeonol (**83**) and ethanol-soluble extractives must be higher than 1.2%, 15.0% respectively, and the moisture and total ash should be no more than 13.0% and 5.0% separately. In comparison, HP ruled that the content of paeonol (**83**) and paeoniflorin (**12**) should not be less than 0.49% and 1.1% respectively, while the JP Sixteenth Edition demands that CM contains not less than 1.0% of paeonol (**83**). In addition, TLC and HPLC assay conditions vary significantly in six pharmacopoeias, too (Table S2). To conclude, current quality criteria of CM are based on a single or a few chemical markers, which fails to reflect the overall quality of CM.

### 5.2. Qualitative and Quantitative Analysis of CM

#### 5.2.1. Thin-Layer Chromatography (TLC) Analysis

TLC analysis is simple, economical and reliable. For reasons of safety, efficacy and quality control, El Babili et al. developed a TLC and microscopic identification technique that systematically studied three species, namely *P. suffruticosa* (tree peony), *Paeonia lactiflora* and *Paeonia veitchii* [119]. This method provides a simple, inexpensive and unambiguous way for establishing the authentication of three similar peony species. Furthermore, when combined with digital scanning and documentation software, TLC provides much more information and parameters. After extraction of CM with ether and ethanol respectively, obtained solutions were separated and analyzed in a TLC solvent system to establish TLC fingerprint, then the TLC plate was scanned under dual wavelength TLC scanner to obtain the quantitative data of characteristic peaks, which subsequently drawn to a column diagram that can intuitively reflect the internal quality of CM [120]. However, the biggest problem of TLC lies in the poor accuracy and low reproducibility.

#### 5.2.2. HPLC Analysis

HPLC analysis for CM usually focuses on phenols, monoterpene glycosides and flavonoids, such as paeonol (**83**), paeonolide (**85**), apiopaeonoside (**91**), gallic acid (**97**), PGG (**70**), paeoniflorin (**12**), oxypaeoniflorin (**13**), catechin (**64**), etc., since these compounds have been proven to exhibit many biological activities and contributes to overall therapeutic effects of CM. The separation was often carried out on reverse-phase C18 columns with binary gradient elution.

Among all the detectors hyphenated to HPLC, UV or DAD are the most commonly applied detectors. Different types of compounds in CM exhibit specific UV absorption characteristics respectively. Monoterpene compounds, often esterified with an aromatic acid such as benzoic acid (**104**), p-hydroxybenzoic (**93**) acid and gallic acid (**97**), expose consistent maximum UV absorption wavelengths with these aromatic acid because neither the pinnae skeleton nor glucose moiety shows UV absorption. Two absorption peaks of flavonoids at 330–360 and 250–270 nm originate from their B and A rings, respectively. Paeonol (**83**) and its derivatives generally display three absorption maxima bands at 225–230, 270–280 and 300–320 nm, respectively [121]. In order to determine various compounds at its peak absorbance wavelength, UV switch methods simultaneously monitoring multiple wavelength were used [122,123]. For example, Ding Yan et al. developed a HPLC method to determine the content of eight pharmacological compounds, namely, gallic acid (**97**), paeoniflorin (**12**), galloylpaeoniflorin (**15**), benzoic acid (**104**), quercetin (**63**), benzoylpaeoniflorin (**17**), paeoniflorigenone (**1**), and paeonol (**83**) [124]. This method was achieved on C18 column by gradient elution with 0.05% formic acid in water and acetonitrile. The method validation gave acceptable linearities (*r* = 0.9996) and recoveries (ranging from 99.4–103.1%). The limits of detection (LOD) of these compounds ranged from 10 to 30 µg/mL.

For the analysis of natural products, chromatographic fingerprint (CFP) techniques, introduced by the World Health Organization (WHO), provide a comprehensive approach that aims to assess the quality of Chinese herbs and their finished products. CM, *Radix Paeoniae Alba*, *Radix Paeoniae Rubra* are important Chinese herbs with similar bioactivities and efficacies. He Chunnian et al. established a HPLC fingerprint method for the quality control of *Radix Paeonia Alba*, *Radix Paeonia Rubra*, and CM, and to compare their main constituents. Eleven chromatographic peaks were identified and differences of chromatographic peaks among these three herbal medicines in chemical compositions were revealed [125]. As we know, due to the different growth environment as well as the processing method, main ingredients contained in CM vary vastly. Wu Meizhen et al. determined chromatographic fingerprints of *P. suffruticosa* by HPLC and applied the clustering analysis for data processing [126]. Results suggest that the quantitative differences among different growing areas could be used to classify herbals from different growing areas, while there seemed to be no quantitative differences for processing factor. Hu Yunfei et al. establish and compare UPLC fingerprint of CM before and after stir-frying, the results show significant differences between fingerprints of CM and charred CM, in which the contents of 5-hydroxymethyl furfural and paeoniflorin (**12**) changed dramatically [127]. This method can reflect the differences of component before and after stir-frying quickly and effectively, and provides the scientific basis for processing technology and quality evaluation of CM. To obtain the characteristic chromatographic profiles of CM, Fan Xuhang et al. developed a UPLC method that determine fifteen batches of CM on an HSS T3 column (2.1 mm × 100 mm, 1.8 μm) eluted with the mobile phase consisted of water containing 0.05% phosphoric acid and acetonitrile in gradient mode with detection wavelength set at 254 nm [128]. The results indicate there were 20 common peaks in the characteristic chromatographic profile of 15 samples, 10 of which were identified, and the similar degrees of the fifteen batches to the common mode were between 0.973–0.998.

Currently, HPLC remains the dominant analytical methods in the routine qualitative or quantitative analysis of CM, due to high reproducibility and sensitivity, good linearity and relatively inexpensive instrument.

#### 5.2.3. LC-MS Analysis

LC-MS has been a powerful analytical tool for the rapid identification of chemical constituents in herbal medicine. It combined the separation of HPLC and the structure information provided by MS which is extremely advantageous in the analysis of complex herbal matrix compared with the conventional arduous and time-consuming phytochemical techniques. Besides, there are some compounds with no UV absorption in CM, such as terpenoids, steroids, fatty acids and sugars, MS detector may be good options for the analysis of these compounds [129]. Meanwhile, high resolution MS, like quadruple time-of-flight mass spectrometry (QTOF-MS), deliver a powerful tool for identification of analytes and mass measurements. HPLC coupled with QTOF-MS can provide valuable information to rapidly quantify the potential chemical markers for herbs with similar chemical characteristics, such as albiflorin (**14**), paeoniflorin (**12**), oxypaeoniflorin (**13**), benzoylpaeoniflorin (**17**), galloylalbiflorin (**15**) and paeoniflorigenone (**1**) [130]. For example, He Qing et al. reported a HPLC-DAD-ESI/MS^n^ method which identify seventeen peaks by their characteristic UV profile and the information of molecular structure provided by ESI/MS^n^ experiments while simultaneously determine five key pharmacological compouds, namely gallic acid (**97**), oxypaeoniflorin (**13**), paeoniflorin (**12**), benzoylpaeoniflorin (**17**), and paeonol (**83**), by the validated HPLC-DAD method. This method, with good linearity, precision and recoveries, combined the chromatographic fingerprints and quantification assay [131]. In addition, capillary high performance liquid chromatography coupled with electrospray ionization mass spectrometry was reported to rapidly analyze pinnae monoterpene glycosides in CM [132].

LC-MS was usually employed to differentiate CM with different processing methods or from different regions and authenticate CM from substitute drugs. Deng Xianmei et al. established a HPLC-DAD-ESIMS method to study the difference of chemical composition between raw and processed CM. Significant changes in their chemical compositions before and after stir-frying processed were detected, which may explain the different medicinal properties of raw and processed CM [133]. Besides, in the sulfur-fumigated CM, the amount of sulfur dioxide was significantly decreased, while sulfur-containing markers, oxypaeoniflorin sulfonate and benzoylpaeoniflorin sulfonate, were not decreased after eight-month storage. Therefore, sulfur dioxide residue index alone may not objectively reflect the sulfur-fumigation extent (quality change extent) of CM. Hence, a more specific method using characteristic sulfur-containing derivatives as chemical makers should be developed to supplement the sulfur dioxide residue determination in the quality control of sulfur-fumigated CM [134]. Again, in some regions, such as Yunnan and Sichuan Provinces in China, root cortex of *P. delavayi* and *P. decomposita* also are used under the name of *P. suffruticosa*. To characterize and differentiate these three species, Xu Shunjun et al. make a comparison of their chemical constituents by HPLC-DAD/ESI-MS^2^. The large differences in chemical compounds among the three *Paeonia* species indicate that galloylglucose and acetophenone patterns could be used as taxonomic markers to differentiate these three *Paeonia* species [121].

As we know, LDP in Chinese pharmacopeia was assessed by the content of two active compounds, paeonol (**83**) from CM and loganin from *Cornus officinalis*, but content determination of only two active compounds cannot fully reflect the holistic quality of LDP. There are many articles reporting HPLC fingerprint of LDP condensed pills, but few articles have identified the common chromatographic peaks due to lack of reference standards. In a recent study, Q-TOF-MS-IDA-MS/MS method was employed for the qualitative determination of eighteen chromatographic peaks without reference standards. By comparing the HPLC chromatographic fingerprints of LDP condensed pills and CM extract, it is confirmed that paeonol (**83**), paeoniflorin (**12**). mudanpioside C (**20**) and oxypaeoniflorin (**8**), galloylpaeoniflorin (**15**), benzoylpaeoniflorin contained in LDP condensed pills come from CM [135].

Metabolomics was initially proposed as a powerful approach for comprehensively profiling endogenous metabolites at a cellular or organ level [136]. LC-MS based metabolomics approaches are being successfully employed in many evaluations of the holistic quality of medicinal herbs. UPLC-QTOF-MS based metabolomics coupled with characteristic ion exploration, a novel and practical strategy was proposed for the rapid evaluation of holistic quality variations caused by the sulfur-fumigation of CM [137]. The results suggested that sulfur-fumigation could significantly affect the holistic quality of CM by chemically transforming pinane monoterpene glucosides, the main bioactive components of CM, to their corresponding sulfonate derivatives. Similarly, Xiao Chaoni et al. proposed a HPLC–MS method to gain deeper insights for revealing metabolomic variations in different root parts of CM in order to enable quality control [138]. The results suggested that the axial roots have higher quality than the lateral roots in CM due to the accumulation of bioactive secondary metabolites associated with plant physiology. Liu Jianhua et al. [139] established a method which combined serum pharmacochemistry with multiple data processing approach to screen the bioactive components and their metabolites in CM by UPLC-MS. The results, obtained from a comprehensive comparative analysis of the fingerprints of the CM and its metabolic fingerprints in rat biological samples, indicated that 23 components in the CM were absorbed into the rat body. In addition, only seven components were found in the metabolic fingerprints, which suggested that they might be metabolites of some components in the CM.

#### 5.2.4. GC Analysis

GC and GC-MS are unanimously accepted methods for the analysis of volatile constituents of TCMs, due to their sensitivity, stability and high efficiency [140]. To analyze the constituents of CM methanol extract from five growing areas (Tongling, Bozhou, Diangjiang, Yuncheng and Jiaxing), GC-MS and NIST05 database were used to analyze the constituents of CM extract, then the result was verified by principal components analysis (PCA) and partial least squares and discriminant analysis (PLS-DA) [141]. Forty-one constituents were identified in CM extract, 21 of which were common constituents. Among them, aromatic and fatty acid compounds were main constituents, accounting for more than 80% of the total extract. In addition, there were some differences in the relative contents and types of chemical constituents of CM from different growing areas.

#### 5.2.5. CE Analysis

Capillary electrophoresis is increasingly important for the quality control of herbal drugs due to its minimum sample and solvents consumption, short analysis time and high separation efficiency [142]. Recent years, the application of CE on the analysis of herbal medicines has become an active area of study. CM contains a series of water-soluble tannins. Wu Yating et al. develop a rapid and efficient method based on HPLC and CE, using a phosphate eluent and a 5C18-MS separating column, successfully analyze eight tannins at detection wavelength of 280 nm. The detection limit for the marker substances varied from 0.04 to 0.93 µg/mL for the HPLC method and 0.02 to 0.36 µg/mL for the CE method [143]. Furthermore, micellar electrokinetic capillary chromatography (MEKC) is a well-established separation mode of CE. The merit of HPLC is also emerged in MEKC, which is particularly useful for the analysis of complex mixture. MEKC and LC were applied to determine paeonol (**83**) and paeoniflorin (**12**) in CM respectively. The optimized buffer system containing 10 mM borate and 25 mM SDS at pH 9.5 were employed. Good linear behavior was exhibited over the investigated concentration range. It was shown that no significant difference was found in the analysis of CM by the developed MEKC and HPLC methods [144].

Electrochemical detection (ECD) typically operated in the amperometric mode can be coupled with CE to provide high sensitivity and selectivity for the determination of electro-active substances [145]. Chen Gang et al. establish a method based on capillary electrophoresis with electrochemical detection for the separation and determination of paeoniflorin (**12**), sucrose, paeonoside (**84**), glucose, and fructose in CM [146,147]. As the primary metabolites, sucrose, glucose, and fructose are found widely presented in plants and higher contents of sugars can indicate the better quality of some herbal drugs [148]. This CE-ECD method is characterized by its higher resolution and sensitivity, lower expense of operation and less amount of sample. Besides, the main advantage of CE as an analytical technique for the analysis of plant samples is that the capillary is much easier to wash.

#### 5.2.6. Spectrometric Methods and Others

Recently, quantitative ^1^H-NMR (^1^H-qNMR) was applied to the determination of paeonol (**83**) concentration in CM, Hachimijiogan, and Keishibukuryogan [149]. The ^1^H-qNMR method has many advantages, it requires neither reference compounds for establishing calibration curves nor sample pre-purification, but it is limited by its inherent low sensitivity. NMR was employed to determine the distribution of metabolites in the root bark of different tree peony cultivars for quality assessment. Sixteen metabolites including sucrose, acetophenones, phenols, monoterpene glycosides, flavonoids and unsaturated fatty acids were simultaneously identified and quantified [150]. Besides, to identify three different samples, CM in Tongling, Luoyang and *P. lactifloral* pall in Hanzhou, Fourier transform infrared (FTIR) spectroscopy combined with second derivative spectra and two-dimensional correlation infrared spectroscopy was applied [151]. Significant difference was found in the two-dimensional spectra in the range of 1730~1380 cm^−1^ and 1000~500 cm^−1^ within the three samples. This result suggests that FTIR combined with 2D correlation IR can be successfully and rapidly applied to distinguish CM among different geographical regions.

Yang Suling et al. developed a simple, highly sensitive method using modified glassy carbon electrode with Nafion/multi-wall carbon nanotubes as a sensitive voltammetric sensor to determine the content of paeonol (**83**) in several pharmaceutical and biological samples, including CM, LDP and paeonol (**83**) spiked LDP, urine, and plasma samples [152]. This modified electrode was characterized by spaghetti-like porous surface, and it significantly increased the oxidation peak current of paeonol (**83**) while reducing the oxidation potential. This method could be successfully applied to the quantification of paeonol (**83**) in drug and biological samples. Papers were published recently about quantum dots (QDs) based fluorescence quenching method to determine constituents like paeonol (**83**) and paeoniflorin (**12**). Semiconductors QDs are used as fluorescence nanosensor because of their high chemical stability and photoluminescence quantum yield. Aqueous polymethylmethacrylate (PMMA)-capped CdSe/ZnS quantum dots were used as fluorescence probes for paeonol (**83**) determination [153]. Additionally, water soluble ZnSe QDs modified by mercaptoacetic acid were used to determinate paeoniflorin (**12**) in aqueous solutions by the fluorescence spectroscopic technique [154]. Compared with the CdSe/ZnS QDs, ZnSe QDs can be directly and simply synthesized in a water-phase system, and the synthetic process is more reproducible and cost effective, and less expensive and toxic.

Besides, Jiang Lei et al. detected the contents of inorganic elements in 15 batches of *P. suffruticosa* from different origins, the result showed that the main elements in CM were lithium, zinc, lead, iron and potassium: the contents of inorganic elements in *P. suffruticosa daodi* and non-*daodi* regional drug showed certain differences [155]. The intricate relationship between therapeutic effects of CM and the morphological and dissolution characteristics of various trace elements in CM need further investigation [156]. Also, chemical analysis of CM was investigated, including the organic components assaying using HPLC and the trace metal elements determination by ICP-MS. The results suggested that the essential metals as well as some metallic pollutants were related to the organic compounds on the basis of their concentrations. This suggests the close relationship between organic and inorganic compounds [157]. Given the special effects of trace elements on the quality of CM, it is suggested that the trace element should be considered and included when establishing chemical fingerprints of CM [158].

In comparison, TLC analysis allows authentication of peonies in a simple, inexpensive and unambiguous way, but TLC was less accurate and often showed less reproducible result between inter-laboratory results. For multi-component quantitative analysis of CM, HPLC and hyphenated techniques are dominant method to be routinely conducted for analyzing CM, due to its easy operation, wide suitability and high accuracy characteristics. GC was usually employed to detect the volatile components, like paeonol (**83**), and pesticide residue in CM. CE showed higher resolution and sensitivity compared to HPLC but displayed poor reproducibility. LC-MS is not only a good choice for identification of unknown compounds but also proper for quantitative analysis with high sensitivity. LC-MS is widely employed to study the chemical profiling of CM in pharmacokinetic studies and in vivo metabolomics research. However, LC-MS is currently still confined to an area of research due to expensive instruments. Spectrometric methods, such as NMR, can provide structural information, so NMR can be a good option with the absence of reference standard. Besides, other methods, like QDs, provide good alternatives in the quality evaluation of CM.

## 6. Safety

CM, as a commonly used TCM, was generally considered safe and showed few adverse drug effects in the clinic use during long history. CM does not contain obvious toxic ingredients [159] except benzoic acid (**104**). Benzoic acid (**104**) was considered to be harmful constituent, but the content of benzoic acid in CM is very low [160]. Moreover, no clinical or biochemical evidence of adverse drug reaction concerning CM was found during literature retrieval. However, TCMs are easily contaminated with heavy metal through polluted soils, irrigation waters, atmospheric dusts, automobile and industrial exhausts, as well as pesticides and fertilizers [161]. Despite its innate safety, CM may be contaminated by exogenous harmful substances, like heavy metals, pesticide residue, or excess in sulfur content by sulfur fumigation. A method combining gas chromatography and matrix solid-phase dispersion was proposed to simultaneously determine 11 pesticide residues in CM, such as organochlorines and pyrethroid [162].

As we know, trace elements play an important role in plant growth and formation of active chemical constituents. It is reported that Cu in soil enhances paeonol (**83**) accumulation in CM of *P. suffruticosa* “Fengdan” [163]. However, some heavy metals (zinc, iron, copper, chromium, and cobalt) may be beneficial at low concentration and become toxic at high concentration, while others (lead and cadmium) have no known beneficial properties and are hence exclusively toxic [164]. Therefore, determinations of trace elements in CM are crucial for understanding the nutritive importance of some elements (Fe, Zn, Cu, Mn) and quality assurance of CM [165]. Chinese Pharmacopoeia (2015 edition) did not specify the determination of heavy metals and other harmful elements, but several researches detected the existence of heavy metals in CM [166]. Planting soil of CM in different producing areas was rich in lead and cadmium, and it is essential to protect planting soil from heavy metal pollution [167].

## 7. Conclusions

TCMs are invaluable resources for new drug discovery, and they are drawing more and more attention worldwide by virtue of their specific theory and long historical clinical practice [140]. CM, as one of the commonly used TCMs, plays important roles in TCM formula or prescription, like LDP, Shuangdan Capsule, Guizhi Fuling Pills, etc. However, unmanageable quality is the bottleneck for its modernization and globalization.

It is now commonly believed that TCMs owe their biological activities to the synergistic effects of all the major and minor components in the medicine. One hundred and nineteen compounds were found from CM, which can be assigned to seven classes: monoterpenes, monoterpene glycosides, flavonoids, tannins, triterpenoids, phenols and others. Many of them are proven to be effective for certain diseases or protein targets, this contributes to the wide range of pharmacological effects of CM, anti-oxidant, anti-inflammatory, anti-tumor, etc. The various structurally complex metabolites in CM might be promising candidates for lead compounds in new drug development. Moreover, omics methods, like proteomics and metabolomics, and network pharmcology investigations of CM should be conducted to unravel the pharmacological mechanisms of CM involving multi-components and multi-targets [168].

Various analytical methods, such as HPLC, CE and LC-MS are capable of determining the content of paeonol (**83**), paeonoflorin (**12**) and other compounds, and simultaneously obtaining chromatographic fingerprints of CM. These methods were used to evaluate CM herb from different localities and pharmaceutical manufacturers [169,170]. However, these analytical methods still need to be further improved and optimized to acquire a robust, comprehensive, rapid, applicable analytical approach for quality evaluation of CM. Quality criteria of CM in different countries and areas need to be improved in order to obtain a more harmonized quality standard. Selection of reference substances is the key point for quality evaluation of herbal products and simultaneous monitoring of multiple components has become a tendency nowadays [171]. Current criteria for CM only include the determination of certain selected constituents of higher content, such as paeonol (**83**) and paeoniflorin (**12**). Some bioactive compounds, such as gallic acid (**97**), paeoniflorin (**12**), benzoylpaeoniflorin (**17**), α-benzoyloxypaeoniflorin (**28**), benzoic acid (**104**) and quercetin (**63**) are frequently chosen to be marker compounds in the authentication and quality evaluation of CM. These compounds should be considered to be new chemical markers in the quality criteria of CM due to their potent pharmacological effects and relatively high contents in CM.

## Supplementary Materials

Supplementary materials are available online.
