# Supplementary material for: Origins, Phytochemistry, Pharmacology, Analytical Methods and Safety of Cortex Moutan (Paeonia suffruticosa Andrew): A Systematic Review
Source: Molecules. 2017 Jun 7;22(6):946. doi: 10.3390/molecules22060946 (PMC6152737; doi:10.3390/molecules22060946)
Supplement: Supplementary file 1 [file molecules-22-00946-s001.pdf]

Table S1 119 chemical compounds isolated from CM.

No.	Compounds	Class	Molecular formula	REF.
**1**	Paeoniflorigenone	Monoterpenes	$C_{17}H_{18}O_6$	[1]
**2**	Paeonisuffral	Monoterpenes	$C_{10}H_{14}O_5$	[2]
**3**	Isopaeonisuffral	Monoterpenes	$C_{10}H_{14}O_5$	[3]
**4**	Paeonisuffrone	Monoterpenes	$C_{10}H_{14}O_4$	[3]
**5**	Deoxypaeonisuffrone	Monoterpenes	$C_{10}H_{14}O_3$	[3]
**6**	(-)-Paeonisuffrone	Monoterpenes	$C_{14}H_{18}O_4$	[4]
**7**	Paeonisothujone	Monoterpenes	$C_{10}H_{14}O_3$	[3]
**8**	Paeoniflorin A	Monoterpenes	$C_{11}H_{14}O_4$	[5]
**9**	Paeoniflorin B.	Monoterpene glycosides	$C_{36}H_{42}O_{17}$	[6]
**10**	3-O-Methylpaeonisuffral	Monoterpenes	$C_{11}H_{16}O_5$	[7]
**11**	6-Methoxypaeoniflorigenone (6-methoxypaeonigenone)	Monoterpenes	$C_{18}H_{20}O_6$	[1]
**12**	Paeoniflorin	Monoterpene glycosides	$C_{23}H_{28}O_{11}$	[4,7]
**13**	Oxypaeoniflorin	Monoterpene glycosides	$C_{23}H_{28}O_{12}$	[4]
**14**	Albiflorin	Monoterpene glycosides	$C_{23}H_{28}O_{11}$	[4]
**15**	Galloylpaeoniflorin	Monoterpene glycosides	$C_{30}H_{32}O_{15}$	[7]
**16**	Galloyloxypaeoniflorin	Monoterpene glycosides	$C_{30}H_{32}O_{16}$	[8]
**17**	Benzoylpaeoniflorin (6'-*O*-Benzoyl Paeoniflorin)	Monoterpene glycosides	$C_{30}H_{32}O_{12}$	[7]
**18**	Mudanpioside A	Monoterpene glycosides	$C_{31}H_{34}O_{13}$	[9]
**19**	Mudanpioside B	Monoterpene glycosides	$C_{31}H_{34}O_{14}$	[10,11]
**20**	Mudanpioside C（Oxybenzoylpaeoniflorin）	Monoterpene glycosides	$C_{30}H_{32}O_{13}$	[9]
**21**	Mudanpioside D（4''-Methoxy-paeoniflorin）	Monoterpene glycosides	$C_{24}H_{30}O_{12}$	[9]
**22**	Mudanpioside E	Monoterpene glycosides	$C_{24}H_{30}O_{13}$	[9]
**23**	Mudanpioside F	Monoterpene glycosides	$C_{16}H_{24}O_8$	[9]
**24**	Mudanpioside G	Monoterpene glycosides	$C_{16}H_{24}O_8$	[12]
**25**	Mudanpioside H	Monoterpene glycosides	$C_{30}H_{32}O_{14}$	[12]
**26**	Mudanpioside I	Monoterpene glycosides	$C_{23}H_{28}O_{11}$	[12]
**27**	Mudanpioside J	Monoterpene glycosides	$C_{31}H_{34}O_{14}$	[4]
**28**	*α*-Benzoyloxypaeoniflorin	Monoterpene glycosides	$C_{30}H_{32}O_{13}$	[13]
**29**	*β*-Benzoyloxypaeoniflorin	Monoterpene glycosides	$C_{30}H_{32}O_{13}$	[13]
**30**	6-*O*-Vanillyoxypaeoniflorin	Monoterpene glycosides	$C_{31}H_{34}O_{15}$	[14]
**31**	Suffrupaeonidanin A	Monoterpene glycosides	$C_{31}H_{34}O_{13}$	[15]
**32**	Suffrupaeonidanin B	Monoterpene glycosides	$C_{31}H_{34}O_{14}$	[15]
**33**	Suffrupaeonidanin C	Monoterpene glycosides	$C_{32}H_{33}O_{13}$	[15]
**34**	Suffrupaeonidanin D	Monoterpene glycosides	$C_{31}H_{34}O_{12}$	[16]
**35**	Suffrupaeonidanin E	Monoterpene glycosides	$C_{32}H_{36}O_{13}$	[16]
**36**	Suffrupaeonidanin F	Monoterpene glycosides	$C_{32}H_{36}O_{13}$	[16]

**37**	Paeonidanin A	Monoterpene glycosides	$C_{31}H_{34}O_{12}$	[6]
**38**	Paeonidanin C	Monoterpene glycosides	$C_{31}H_{34}O_{13}$	[5]
**39**	Suffruyabiosides A	Monoterpene glycosides	$C_{36}H_{42}O_{18}$	[17]
**40**	Suffruyabiosides B	Monoterpene glycosides	$C_{36}H_{42}O_{17}$	[17]
**41**	Paeoniside A	Monoterpene glycosides	$C_{30}H_{32}O_{12}$	[18]
**42**	Paeoniside B	Monoterpene glycosides	$C_{30}H_{32}O_{15}$	[18]
**43**	Suffrupaeoniflorin A	Monoterpene glycosides	$C_{36}H_{42}O_{18}$	[16]
**44**	Suffrupaeoniflorin B	Monoterpene glycosides	$C_{31}H_{34}O_{13}$	[16]
**45**	Oxypaeonidanin	Monoterpene glycosides	$C_{24}H_{30}O_{12}$	[6]
**46**	9-Epi-oxypaeonidanin	Monoterpene glycosides	$C_{24}H_{30}O_{12}$	[6]
**47**	8-O-benzoylpaeonidanin	Monoterpene glycosides	$C_{31}H_{34}O_{12}$	[19]
**48**	9-O-butyloxypaeonidanin	Monoterpene glycosides	$C_{27}H_{36}O_{12}$	[6]
**49**	9-O-butylpaeonidanin	Monoterpene glycosides	$C_{27}H_{36}O_{11}$	[6]
**50**	*p*-Hydroxylbenzoylpaeonidanin	Monoterpene glycosides	$C_{31}H_{34}O_{13}$	[6]
**51**	4-O-methylsuffrupaeoniflorin B	Monoterpene glycosides	$C_{32}H_{36}O_{13}$	[16]
**52**	Paeoniflorin-4-ethyl ether	Monoterpene glycosides	$C_{25}H_{32}O_{11}$	[20]
**53**	4-O-butylpaeoniflorin	Monoterpene glycosides	$C_{27}H_{36}O_{11}$	[19]
**54**	4-O-methylmoudanpioside C	Monoterpene glycosides	$C_{31}H_{34}O_{13}$	[5]
**55**	4-O-methylbenzoyloxypaeoniflorin	Monoterpene glycosides	$C_{31}H_{34}O_{13}$	[6]
**56**	Oxypaeoniflorin sulfonate.	Monoterpene glycosides	$C_{23}H_{27}O_{11}$	[6]
**57**	4-O-methyloxypaeoniflorin	Monoterpene glycosides	$C_{24}H_{30}O_{12}$	[6]
**58**	4-O-methylgalloyloxypaeoniflorin	Monoterpene glycosides	$C_{31}H_{34}O_{16}$	[6]
**59**	4-O-butyloxypaeoniflorin.	Monoterpene glycosides	$C_{27}H_{36}O_{12}$	[6]
**60**	4-O-methylpaeoniflorin	Monoterpene glycosides	$C_{24}H_{30}O_{11}$	[6,18]
**61**	8-O-debenzoylpaeoniflorin	Monoterpene glycosides	$C_{16}H_{24}O_{10}$	[6]
**62**	4-O-methylbenzoylpaeoniflorin	Monoterpene glycosides	$C_{31}H_{34}O_{12}$	[6]
**63**	Quercetin	Flavonoids	$C_{15}H_{10}O_7$	[4]
**64**	Catechin	Flavonoids	$C_{15}H_{14}O_6$	[4,21]
**65**	Kaempferol	Flavonoids	$C_{15}H_{10}O_6$	[4]
**66**	Flavan-3-ols catechin	Flavonoids	$C_{15}H_{14}O_6$	[22]
**67**	Epicatechin-3-O-gallate	Flavonoids	$C_{22}H_{18}O_{10}$	[22]
**68**	Dimeric proanthocyanidin epicatechin-(4*β* → 8)-catechin	Flavonoids	$C_{30}H_{26}O_{12}$	[22]
**69**	(+)-Catechin-7-O-*β*-glucopyranoside	Flavonoids	$C_{22}H_{26}O_{10}$	[23]
**70**	1,2,3,4,6-Penta-*O*-galloyl-*β*-D-glucose	Tannins	$C_{41}H_{32}O_{26}$	[21]
**71**	Trigalloyl-glucoses	Tannins	$C_{27}H_{24}O_{18}$	[22]
**72**	(-)-Epigallocatechin gallate	Tannins	$C_{22}H_{18}O_9$	[24]
**73**	*β*-Sitosterol	Triterpenoids	$C_{29}H_{50}O$	[25]
**74**	Daucosterol (*β*-sitosterol-*β*-D-glucoside)	Triterpenoids	$C_{35}H_{60}O_6$	[25]
**75**	Campesterol	Triterpenoids	$C_{28}H_{48}O$	[25]
**76**	Palbinone	Triterpenoids	$C_{22}H_{30}O_4$	[25]
**77**	Hederagenin	Triterpenoids	$C_{30}H_{48}O_4$	[18]
**78**	Oleanolic acid	Triterpenoids	$C_{30}H_{48}O_3$	[18]

**79**	Betulinic acid	Triterpenoids	$C_{30}H_{48}O_3$	[25]
**80**	Mudanpinoic acid A	Triterpenoids	$C_{30}H_{46}O_3$	[26]
**81**	30-Norhederagenin	Triterpenoids	$C_{29}H_{44}O_4$	[1]
**82**	Ursolic acid	Triterpenoids	$C_{30}H_{48}O_3$	[25]
**83**	Paeonol	Phenols	$C_9H_{10}O_3$	[27]
**84**	Paeonoside	Phenols	$C_{15}H_{20}O_8$	[4,27]
**85**	Paeonolide	Phenols	$C_{20}H_{28}O_{12}$	[8]
**86**	Suffruticoside A	Phenols	$C_{27}H_{32}O_{16}$	[8]
**87**	Suffruticoside B	Phenols	$C_{27}H_{32}O_{16}$	[8]
**88**	Suffruticoside C	Phenols	$C_{27}H_{32}O_{16}$	[8]
**89**	Suffruticoside D	Phenols	$C_{27}H_{32}O_{16}$	[8]
**90**	Suffruticoside E	Phenols	$C_{26}H_{38}O_{17}$	[8]
**91**	Apiopaeonoside	Phenols	$C_{20}H_{28}O_{12}$	[4]
**92**	Phenol	Phenols	C_6H_6O	[4]
**93**	*p*-Hydroxybenzoic acid	Phenols	$C_7H_6O_3$	[4]
**94**	2,5-Dihydroxy-4-methoxyacetophenone	Phenols	$C_8H_8O_3$	[27]
**95**	Methyl 3-hydroxy-4-methoxybenzoate	Phenols	$C_9H_{10}O_4$	[27]
**96**	*trans*-Caffeic acid stearyl ester	Phenols	$C_{27}H_{44}O_4$	[26]
**97**	Gallic acid	Phenols	$C_7H_6O_5$	[4]
**98**	Me gallate (Gallicin)	Phenols	$C_8H_8O_5$	[4]
**99**	Gallacetophenone	Phenols	$C_8H_8O_4$	[24]
**100**	Mudanoside A	Phenols	$C_{14}H_{18}O_9$	[12]
**101**	Mudanoside B	Phenols	$C_{18}H_{24}O_{14}$	[26]
**102**	Mudanoside C	Phenols	$C_{15}H_{20}O_9$	[28]
**103**	Acetovanillone	Phenols	$C_9H_{10}O_3$	[7]
**104**	Benzoic acid	Phenols	$C_7H_6O_2$	[4]
**105**	*p*-Hydroxyacetophenone	Phenols	$C_{19}H_{26}O_{12}$	[4]
**106**	3-Hydroxy-4-methoxybenzoic acid	Phenols	$C_8H_8O_4$	[4]
**107**	Resacetophenone	Phenols	$C_8H_8O_3$	[4]
**108**	2,3-Dihydroxy-4-methoxyacetophenone	Phenols	$C_9H_{10}O_4$	[4]
**109**	3-Hydroxy-4-methoxyacetophenone	Phenols	$C_9H_{10}O_3$	[4]
**110**	3-O-D-glucopyranosyl-4-methoxyacetophenone	Phenols	$C_{15}H_{20}O_8$	[28]
**111**	Iriflophenone 2-O-β-D-glucopyranoside	Phenols	$C_{19}H_{20}O_{10}$	[28]
**112**	Adenosine	Others	$C_{10}H_{13}N_5O_4$	[4]
**113**	Uridine	Others	$C_{19}H_{14}N_2O_6$	[23]
**114**	1-Tryptophan	Others	$C_{11}H_{12}N_2O_2$	[23]
**115**	Thymidine	Others	$C_{10}H_{16}N_2O_5$	[23]
**116**	Ainsliaside E	Others	$C_{21}H_{38}O_9$	[23]
**117**	Paesuffrioside	Others	$C_{14}H_{21}NO_7$	[23]
**118**	Oxo-acetic acid 2-ethoxy-4-(3-hydroxy-2-oxopropyl) phenyl ester	Others	$C_{13}H_{14}O_6$	[29]
**119**	5-Hydroxy-3S-hydroxymethyl-6-methyl-2,3-dihydrobenzofuran	Others	$C_{10}H_{12}O_3$	[1]

Table S2 Comparative table of quality criteria of CM in pharmacopoeias of different countries(areas): Japanese pharmacopoeia (JP), Chinese pharmacopoeia (CP), Korean pharmacopoeia (KP), Vietnamese Pharmacopoeia (VP), Hong Kong Chinese Materia Medica Standards (HP) and Taiwan Herbal Pharmacopoeia(TP)

1. Names of CM in JP, CP, KP, VP, HP and TP

Paeonia suffruticosa Andrews		English titile	Part of use
JP	MOUTAN CORTEX<br>*Paeonia suffruticosa* Andrews<br>(*Paeonia moutan* Sims)	Moutan Bark	root bark
CP	CORTEX MOUTAN<br>*Paeonia suffruticosa* Andr.	Tree Peony Bark	root bark (removed rootlets)
KP	MOUTAN CORTEX RADICIS<br>*Paeonia suffruticosa* Andrews（*Paeonia moutan* Sims）	Moutan Root Bark	root
VP	CORTEX PAEONIA SUFFURUTICOSAE<br>*Paeonia suffruticosa* Andr.	CORTEX PAEONIAE SUFFRUTICOSAE	root bark
HP	Cortex Moutan<br>*Paeonia suffruticosa* Andr.	Tree Peony Bark	root bark (removed rootlets)
TP	MOUATN RADICS CORTEX<br>*Paeonia suffruticosa* Andr.	Tree Peony Bark	root bark

2. Description of CM in JP, CP, KP, VP, HP and TP

Paeonia suffruticosa Andrews	Title	Latin title	length	diameter	Thickness	microscope powder
JP	ボタンピ	MOUTAN CORTEX	5-8cm	0.8-1.5cm	0.5cm	●
CP	牡丹皮	CORTEX MOUTAN	5-20cm	5-12mm	1-4mm	●
KP	목단피	MOUTAN CORTEX RADICIS	5-8cm	5-12mm	1-4mm	
VP	Mẫu đơn bì	CORTEX PAEONIAE SUFFRUTICOSAE	5-20cm	0.5-1.2cm	0.1-0.4cm	●
HP	牡丹皮	CORTEX MOUTAN	<20cm	5-16mm	1-4.5 mm	●
TP	牡丹皮	MOUTAN RADICIS CORTEX	5-25cm	0.5-1.4cm	2-4mm	

2. Testing methods and specification values for CM in JP, CP, KP, VP, HP and TP

Latin name		Identification	Purification	Loss on drying	Total ash	Acid insoluble ash	Extract content	Assay
CP	CORTEX MOUTAN	O (TLC)	X	O (≯13.0%, Water)	O (≯5.0%)	O (≯1.0%)	≮ 15.0% (Ethanol-soluble extract)	Paeonol ≮ 1.2% (HPLC)
JP	MOUTAN CORTEX	O (TLC)	O (Xylem, Foreign matter)	X	O (≯6.0%)	O (≯ 1.0%)	X	Paeonol ≮ 1.0% (HPLC)
KP	MOUTAN CORTEX RADICIS	O (TLC)	O (Xylem, Foreign matter)	X	O(≯ 6.0%)	O (≯1.0%)	X	Paeonol ≮ 1.0% (HPLC)

VP	CORTEX PAEONIAE SUFFRUTICOSAE	O (TLC)	O (Wood, Foreign matter)	O (≯13.0%)	O (≯5.0%)	X	X	Paeonol ≮ 1.2% (Absorption)
HP	CORTEX MOUTAN	O (TLC)	X	O (≯13.0%, Water)	O (≯5.0%)	O (≯1.0%)	≮ 18.0% (Ethanol-soluble extract)	Paeonol ≮ 1.1% (HPLC) Paeoniflorin ≮0.49% (HPLC)
TP	MOUTAN RADICIS CORTEX	O (TLC)	X	O (≯13.0%, Water)	O (≯5.0%)	O (≯ 1.0%)	X	Paeonol ≮ 1.0% (HPLC) Paeoniflorin ≮0.5% (HPLC)

4. TLC Conditions of Identification for CM in JP, CP, KP, VP, HP and TP

Latin name		TLC condition (1) developing solvent	(2) detection	(3) color tone on TLC	(4) marker compounds
CP	CORTEX MOUTAN	cyclohexane / ethyl acetate (3 : 1)	5% frerric chloride in ethanol (acidifide with HCl)	bluish-brown	paeonol
JP	MOUTAN CORTEX	hexane / ethyl acetate (1 : 1)	UV 254 nm		paeonol
KP	MOUTAN CORTEX RADICIS	hexane / ethyl acetate (1 : 1)	UV 254 nm		paeonol
VP	CORTEX PAEONIAE SUFFRUTICOSAE	cyclohexane / ethyl acetate (3 : 1)	5% frerric chloride in ethanol		paeonol
HP	CORTEX MOUTAN	Chloroform/acetic ether/Methanol/ formic acid(40:5:10:0.2)	50%(v/v) dilute sulphuric acid and 2%(w/v) p-hydroxyl benzaldehyde methanol solution	Same color with reference solution	Paeonol paeoniflorin
TP	MOUTAN RADICIS CORTEX	hexane / ethyl acetate (3 : 1)	UV 254 nm		paeonol

5. Assay conditions for CM in JP, CP, KP, VP, HP and TP

Latin name		Assay (↑ : Not less than)	(1) method	(2) developing solvent	(3) detection
CP	CORTEX MOUTAN	Paeonol ↑ 1.2%	HPLC (ODS column)	methanol / water (45 : 55)	UV 274 nm
JP	MOUTAN CORTEX	Paeonol ↑ 1.0%	HPLC (ODS column, I.D. 4-6 mm x 15-25 cm, 5-10μm)	1) water / acetonitrile / acetic acid (100) (65 : 35 : 2) 2) 20° 3) adjust flow rate to elute paeonol at ca.14 min	UV 274 nm
KP	MOUTAN CORTEX RADICIS	Paeonol ↑ 1.0%	HPLC (ODS column, I.D. 4-6 mm x 15-25 cm, 5-10 μm)	1) water / acetonitrile / acetic acid (100) (65 : 35 : 2) 2) 20° 3) adjust flow rate to elute paeonol at ca.14 min	UV 274 nm
VP	CORTEX PAEONIAE SUFFRUTICOSAE	Paeonol ↑ 1.0%	Absorption	water	UV 274 nm

HP	CORTEX MOUTAN	Paeonol ↑ 1.1%<br>Paeonniflorin ↑ 0.49%	HPLC (ODS column, I.D. 4-6 mm x<br>15-25 cm, 5-10 μm)	Gradient elution with water and acetonitrile	UV 230 nm
TP	MOUTAN RADICIS CORTEX	Paeonol ↑ 1.0%<br>Paeonniflorin ↑ 0.5%	HPLC (ODS column, I.D.4.6 x250mm, 5μm)	Water/acetonitrile/acetic acid(65:35:2) for paeonol; water/acetonitrile(4:1) for paeoniflorin	UV 274 nm (paeonol)<br>UV 230 nm (paeoniflorin)

PS: (●:have, O: Established, X: Not established, : ≯Not more than, : ≮Not less than)

1. Ha, D.T.; Ngoc, T.M.; Lee, I. Inhibitors of aldose reductase and formation of advanced glycation end-products in moutan cortex (paeonia suffruticosa). *J. Nat. Prod.* **2009**, *8*, 1465-1470.
2. Yoshikawa, M.; Ohta, T.; Kawaguchi, A.; Matsuda, H. Bioactive constituents of chinese natural medicines. V. Radical scavenging effect of moutan cortex. (1): Absolute stereostructures of two monoterpenes, paeonisuffrone and paeonisuffral. *Chemical & pharmaceutical bulletin* **2000**, *48*, 1327-1331.
3. Yoshikawa, M.; Harada, E.; Minematsu, T. Absolute stereostructures of paeonisothujone, a novel skeletal monoterpene ketone, and deoxypaeonisuffrone, and isopaeonisuffral, two new monoterpenes, from mouan cortex. *Chem. Pharm. Bull* **1994**, *42*, 736-738.
4. He, C.N.; Peng, Y.; Zhang, Y.C.; al., e. Phytochemical and biological studies of paeoniaceae. *CHEMISTRY & BIODIVERSITY* **2010**, *7*.
5. Ding, L.; Jiang, Z.; Liu, Y.; Chen, L.; Zhao, Q.; Yao, X.; Zhao, F.; Qiu, F. Monoterpenoid inhibitors of no production from paeonia suffruticosa. *Fitoterapia* **2012**, *83*, 1598-1603.
6. Ding, L.; Zhao, F.; Chen, L.; Jiang, Z.; Liu, Y.; Li, Z.; Qiu, F.; Yao, X. New monoterpene glycosides from paeonia suffruticosa andrews and their inhibition on no production in lps-induced raw 264.7 cells. *Bioorg Med Chem Lett* **2012**, *22*, 7243-7247.
7. Shao-Hua Wu; Da-Gang Wu; Chen, Y.-W. Chemical constituents and bioactivities of plants from the genus paeonia. *CHEMISTRY & BIODIVERSITY* **2010**, *7*, 90-104.
8. Matsuda, H.; Ohta, T.; Kawaguchi, A.; Yoshikawa, M. Bioactive constituents of chinese natural medicines. Vi. Moutan cortex.(2): Structures and radical scavenging effects of suffruticosides a, b, c, d, and e and galloyl-oxypaeoniflorin. *Chemical and pharmaceutical bulletin* **2001**, *49*, 69-72.
9. Lin, H.C.; Ding, H.Y.; Wu, T.S.; Wu, P.L. Monoterpene glycosides from paeonia suffruticosa. *Phytochemistry* **1996**, *41*, 237-242.
10. HANG-CHING LIN; HSIOU-YU DING; TIAN-SHUNG WU; WU., P.-L. Monoterpne glycosides from paeonia suffruticosa. *Phytochemisty* **1995**, *41*, 237-242.
11. Wu, M.G.; Gu, Z.Y. Screening of bioactive compounds from moutan cortex and their anti-inflammatory activities in rat synoviocytes. *Evidence-based complementary and alternative medicine : eCAM* **2009**, *6*, 57-63.
12. Hsiou-Yu, D. Glycosides from paeonia suffruticosa. *Chem, Pharm, Bull* **1999**.
13. Zhou, S.L.; Zou, X.H.; Zhou, Z.Q.; Liu, J.; Xu, C.; Yu, J.; Wang, Q.; Zhang, D.M.; Wang, X.Q.; Ge, S., *et al.* Multiple species of wild tree peonies gave rise to the 'king of

flowers', paeonia suffruticosa andrews. *Proceedings. Biological sciences / The Royal Society* **2014**, *281*.
14. Ren-Bo An , H.-C.K., Sung-Hee Lee. A new monoterpene glycoside and antibacterial monoterpene glycosides from paeonia suffruticosa. **2006**.
15. Yang, Y.; Hu, H.Y.; Yu, N.J.; Zhang, Y.; Zhao, Y.M. Three new paeonidanin-type monoterpene glycosides from paeonia suffruticosa andr. *Helvetica Chimica Acta* **2010**, *93*, 1622-1627.
16. Song, W.H.; Cheng, Z.H.; Chen, D.F. Anticomplement monoterpenoid glucosides from the root bark of paeonia suffruticosa. *Journal of natural products* **2014**, *77*, 42-48.
17. Furuya, R.; Hu, H.; Zhang, Z.; Shigemori, H. Suffruyabiosides a and b, two new monoterpene diglycosides from moutan cortex. *Molecules (Basel, Switzerland)* **2012**, *17*, 4915-4923.
18. Zhu, X.; Fang, Z.H. New monoterpene glycosides from the root cortex of paeonia suffruticosa and their potential anti-inflammatory activity. *Natural Product Research* **2014**, *28*, 301-305.
19. Ha, D.T.; Trung, T.N.; Hien, T.T.; Dao, T.T.; Yim, N.; Ngoc, T.M.; Oh, W.K.; Bae, K. Selected compounds derived from moutan cortex stimulated glucose uptake and glycogen synthesis via ampk activation in human hepg2 cells. *J. Ethnopharmacol.* **2010**, *131*, 417-424.
20. WANG, S.J.; YANG, Y.C.; LI, S. A new paeoniflorin derivative isolated from the root bark ethanol extract of paeonia suffruticosa. *China Journal of Chinese Materia Medica* **2005**, *30*, 759-761.
21. Satoh, K.; Nagai, F.; et al. Inhibition of Na+, K+-ATPase by 1, 2, 3, 4, 6-Penta-O-galloyl-β-D-glucose, a major constituent of both moutan cortex and paeoniae radix. *Biochemical pharmacology* **1997**, 53, 611-614.
22. Wang, R.; Lechtenberg, M.; Sendker, J.; Petereit, F.; Deters, A.; Hensel, A. Wound-healing plants from tcm: In vitro investigations on selected tcm plants and their influence on human dermal fibroblasts and keratinocytes. *Fitoterapia* **2013**, *84*, 308-317.
23. Xiao, K.; Song, Q.H.; Zhang, S.W.; Xuan, L.J. A pyrrole derivative from paeonia suffruticosa. *Natural Product Research* **2008**, *22*, 1614-1619.
24. Lee, S.-J.; Lee, I.-S.; Mar, W. Inhibition of inducible nitric oxide synthase and cyclooxygenase-2 activity by 1,2,3,4,6-penta-o-galloyl-j3-d-glucose in murine macrophage cells. *Archives of pharmacal research* **2003**, *26*, 832-839.
25. Ha Do, T.; Tuan Dao, T.; Thu Nguyen, B.; Nhiem Nguyen, X.; Ngoc Tran, M.; Yim, N.; Bae, K. Palbinone and triterpenes from moutan cortex (paeonia suffruticosa, paeoniaceae) stimulate glucose uptake and glycogen synthesis via activation of ampk in insulin-resistant human hepg2 cells. *Bioorg Med Chem Lett* **2009**, *19*, 5556-5559.
26. Lin, H.C.; Ding, H.Y.; Wu, Y.C. Two novel compounds from paeonia suffructicosa. *J. Nat. Prod.* **1998**, 343-346.
27. Gao Li , C.-S.S., Kyeung-Seon Lee. Protective constituents against sepsis in mice from the root cortex of paeonia suffruticosa. *Archives of pharmacal research* **2004**, *27*, 1123-1126,.
28. Ding, L.; Zuo, Q.; Li, D.; Feng, X.; Gao, X.; Zhao, F.; Qiu, F. A new phenone from the

roots of paeonia suffruticosa andrews. *Nat Prod Res* **2016**, 1-8.

29. Choi, Y.H.; Yoo, H.J.; Noh, I.C.; Lee, J.M.; Park, J.W.; Choi, W.S.; Choi, J.H. Bioassay-guided isolation of novel compound from paeonia suffruticosa andrews roots as an il-1beta inhibitor. *Archives of pharmacal research* **2012**, *35*, 801-805.
